# Lights and Shadows about the Effectiveness of IVF in HIV Infected Women: A Systematic Review

**DOI:** 10.1155/2015/517208

**Published:** 2015-12-08

**Authors:** Catarina Marques, Cristina Guerreiro, Sérgio Reis Soares

**Affiliations:** ^1^Maternidade Dr. Alfredo da Costa, Rua Viriato, 1069-089 Lisboa, Portugal; ^2^Fetal Maternal Department, Maternidade Dr. Alfredo da Costa, Lisbon, Portugal; ^3^Instituto Valenciano de Infertilidade (IVI-Lisboa), Lisbon, Portugal

## Abstract

*Background*. HIV infected women have higher rates of infertility.* Objective*. The purpose of this literature review is to evaluate the effectiveness of fresh IVF/ICSI cycles in HIV infected women.* Materials and Methods*. A search of the PubMed database was performed to identify studies assessing fresh nondonor oocyte IVF/ICSI cycle outcomes of serodiscordant couples with an HIV infected female partner.* Results and Discussion*. Ten studies met the inclusion criteria. Whenever a comparison with a control group was available, with the exception of one case, ovarian stimulation cancelation rate was higher and pregnancy rate (PR) was lower in HIV infected women. However, statistically significant differences in both rates were only seen in one and two studies, respectively. A number of noncontrolled sources of bias for IVF outcome were identified. This fact, added to the small size of samples studied and heterogeneity in study design and methodology, still hampers the performance of a meta-analysis on the issue.* Conclusion*. Prospective matched case-control studies are necessary for the understanding of the specific effects of HIV infection on ovarian response and ART outcome.

## 1. Introduction

The human immunodeficiency virus (HIV) epidemic arose from zoonotic infections with simian immunodeficiency viruses from African primates. Since then, the global epidemiology of HIV infection has changed markedly: the prevalence of HIV has increased from 31 million in 2002 to 36.9 million in 2014, essentially due to prolonged survival caused by antiretroviral therapy, whereas the global incidence has decreased from 3.3 million in 2002 to 2 million in 2014 [1].

Since the introduction of antiretroviral therapies, two major medical achievements have been made, allowing many couples with an HIV positive partner to consider pregnancy planning:

1. Life expectancy of infected patients as well as their life quality has dramatically improved during the last 10 years [2].
2. A significant reduction in mother-to-child HIV transmission (MTCT) has been observed, especially in developed countries, with transmission rates lower than 1% to 2%, compared to 14% to 42% without any intervention. This has been achieved with the use of antiretroviral drug combinations during pregnancy and labor/delivery, neonatal prophylaxis, elective caesarean delivery, and avoidance of breast feeding [3].

Over 80% of people infected with HIV are of reproductive age (15 to 44 years old). Reports suggest that there are currently more than 140,000 HIV serodiscordant heterosexual couples in the United States (US), approximately 50% of whom having reproductive plans [4]. According to the National Perinatal HIV Hotline and Clinicians Network, calls pertaining to HIV serodiscordant couples and their options for safer conception have increased significantly between 2006 and 2011 [5].

Managing HIV infected patients with a childbearing wish involves a multidisciplinary approach, ideally including maternal-fetal medicine specialists, HIV/AIDS specialists, neonatologists, pediatricians, psychiatrists, social workers, and reproductive endocrinologists [6]. Preconception counseling is highly recommended among HIV serodiscordant and seroconcordant couples, allowing them to make more informed choices in order to reduce sexual transmission and improving pregnancy outcome [7]. However, a recent survey of HIV infected women who had been or were pregnant at the time of the questionnaire showed that more than half of them did not have preconception counseling [8]. Evaluating the need for antiretroviral therapy should be part of the initial assessment of preconception counseling. Any concurrent sexually transmitted infection should be treated and safe sexual practices should be encouraged [9].

Infertility affects approximately 15% of the general population and HIV infected patients, both men and women, have higher rates of infertility than their HIV negative counterparts [10, 11].

In serodiscordant couples in which the male partner is infected, assisted reproductive technology (ART) is the safest way to prevent sexual transmission. After the sperm-washing (SW) procedure, there are two main options to achieve a pregnancy: intrauterine insemination (IUI) and in vitro fertilization (IVF)/intracytoplasmic sperm injection (ICSI). In couples with a normal fertility evaluation, IUI is an effective approach. If semen analysis is abnormal, then IVF/ICSI is undoubtedly the treatment to be offered [6]. SW eliminates round cells, seminal plasma, and the majority of immotile sperm. Spermatozoa are isolated by sequential density gradient and swim-up techniques and are subsequently tested by PCR assays for the presence of HIV RNA [12]. A systematic review and meta-analysis summarized the experience with serodiscordant couples with an infected male partner until 2013, with 2,393 SW-IUI and 780 SW-IVF treatment cycles documented [13]. The authors concluded that HIV infected men with noninfected partners have pregnancy and live birth rates with ART comparable to seronegative couples.

In serodiscordant couples in which the female partner is infected, pregnancy can be achieved without the risk of sexual transmission by self-insemination around the time of ovulation [6]. If conception does not occur after more than six cycles of self-insemination, or if a preexisting fertility problem was diagnosed, the use of ART should be envisaged [7].

Most of the reports so far published on IUI or IVF treatments performed in serodiscordant couples refer to infected male partners. Very few studies have addressed IVF outcome in serodiscordant couples with an HIV infected female partner. The purpose of this review is to evaluate the effectiveness of fresh nondonor oocyte IVF/ICSI treatments performed in this population. Control over variables that are traditionally known to influence IVF/ICSI outcome, such as female age, ovarian reserve, race/ethnicity, Body Mass Index (BMI), tobacco consumption, the presence of tubal disease, and the number of embryos replaced in the uterus, was ascertained in the studies found.

## 2. Materials and Methods

**Figure 1:**
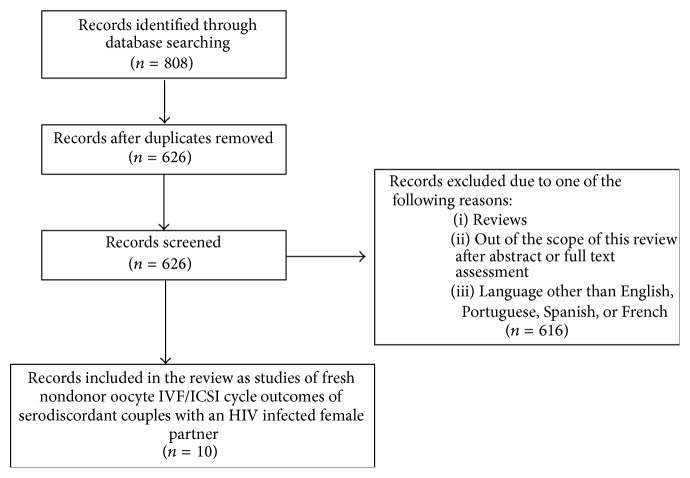
Flow chart for the search methodology.

A search of the PubMed database was performed in order to identify all studies involving ART including the HIV infected population published until July 2014. The search terms used were “HIV” AND “assisted reproduction,” “HIV” AND “assisted reproductive technology,” “HIV” AND “in vitro fertilization,” and “HIV” AND “infertility”. Abbreviations such as “IVF” and “ICSI” were also used. An initial list of 626 studies was obtained. Inclusion criterion was studies assessing fresh nondonor oocyte IVF/ICSI cycle outcomes of serodiscordant couples with an HIV infected female partner. References with abstracts that demonstrated them to be unrelated to the IVF/ICSI cycle outcomes of serodiscordant couples with an HIV infected female partner were excluded without full text assessment, as were reviews and case reports. All original articles with abstracts that indicated them to be within the scope of this study were fully assessed; when this assessment was confirmed, they were included in the review. Articles in languages other than English, Portuguese, Spanish, or French were excluded. Ten studies were finally included.  Figure 1 summarizes the steps involved in literature selection based on Preferred Reporting Items for Systematic Reviews and Meta-Analyses (PRISMA) guidelines [24].

## 3. Results

Studies analyzed reported on ART treatments performed in a total of 342 HIV infected women, with a mean age of 35.4 years, who underwent 516 IVF/ICSI cycles (Table 1). The average CD4 count ranged from “>200” to 712 cells/mm^3^, 48% to 100% of patients in each study had undetectable viral loads, and 44% to 95% of them were being treated with combined antiretroviral therapy.

Table 1 shows baseline characteristics of study and control groups and Table 2 shows the outcomes of ovarian stimulation and IVF.

Among the studies included in this review, data concerning ovarian response to stimulation in HIV infected patients can be summarized as follows: some of the initial studies report the need of higher doses of gonadotropin to achieve satisfactory ovarian response (Terriou et al. 2005 [16]; Coll et al. 2006 [17]), while a normal response to stimulation is described in infected women who are in good general health conditions and reach egg pick-up (Martinet et al. 2006 [18]). Data from most recent studies suggest that a normal ovarian response is seen in these patients (Manigart et al. 2006 [19], Douglas et al. 2009 [20], Prisant et al. 2010 [21], Santulli et al. 2011 [22], and Nurudeen et al. 2013 [23]) (Table 2). However, in the nine studies that assessed the cancellation rate of ovarian stimulation in HIV infected patients, although significance was observed in only one of them (15.2% versus 4.9% in the control group), in all the instances in which a comparison could be made cancellation rate was higher in the study group than in controls (Table 2).

In study groups, the clinical PR per stimulation cycle initiated varied from 6.7% to 24.1% and the clinical PR per embryo transfer varied from 9.1% to 63% (Table 2). Unfortunately, not all studies mentioned the rate per cycle initiated. A summary of the conclusions of the six studies that compared the PRs in HIV infected women with those from noninfected controls is (Table 2) as follows:

- In two studies, HIV infected women had a statistically significantly lower PR (Ohl et al. 2003 [14], Coll et al. 2006 [17]).
- In three studies, the PR of HIV infected women was not statistically significantly different from that of control subjects, but lower values were observed (Martinet et al. 2006 [18], Santulli 2011 et al. [22], and Nurudeen et al. 2013 [23]).
- In one study, the PR of HIV infected women was not statistically significantly different from that of control subjects, but a higher value was observed (Prisant et al. 2010 [21]).

In all studies, PR was reported per embryo transfer, with the exception of two cases of PR per cycle initiated [16, 19] and one of PRs per oocyte retrieval [22]. Noteworthy, vertical transmission of HIV infection was zero.

Concerning variables that are traditionally known to influence IVF/ICSI outcome, data from studies can be summarized as follows.

### 3.1. Female Age

Seven studies included in this review were age-matched. In only one study, ART results were stratified by age: in both age groups (<35 and ≥35 years), infected and control patients had similar IVF/ICSI clinical outcomes with similar clinical PRs per embryo transfer [23].

### 3.2. Ovarian Reserve

Six studies included in this review evaluated the ovarian reserve, but only five of them had a control group (Table 1):

(i) In one of them, the comparison between early follicular phase serum FSH of HIV infected women and controls showed a statistically significant difference (9.0 ± 2.4 versus 7.0 ± 2.9 IU/L, resp., *p* < 0.001) (Ohl et al. 2003 [14]).
(ii) In the other four studies, HIV infected women and controls had similar values for markers of ovarian reserve. FSH and HAM levels and antral follicle count (AFC) were evaluated (Terriou et al. 2005 [16]; Douglas et al. 2005 [20]; Santulli et al. 2011 [22]; Nurudeen et al. 2013 [23]).

### 3.3. Race/Ethnicity

In our review, only two studies documented patients' race/ethnicity [18, 23]. In both of them, the proportion of black women was significantly higher in the study group and this was mentioned as a possible source of bias.

### 3.4. Body Mass Index (BMI)

Only two of the studies analyzed evaluated BMI (Table 1):

(i) In one of them, HIV infected women had higher BMI than control subjects (24.2 versus 22.9, *p* = 0.032) (Santulli et al. 2011 [22]).
(ii) In the other one, HIV infected women and controls had similar BMI (Nurudeen et al. 2013 [23]).

### 3.5. Tobacco

Of the studies analyzed in this review, only two controlled for tobacco consumption:

(i) In the first of them, no differences were found between HIV infected women and controls (Santulli et al. 2011 [22]).
(ii) In the other one, in the group of women ≥35 years of age, infected patients smoked significantly more often than controls (16% versus 0%; *p* < 0.05). Although parameters of ovarian reserve, the number of mature oocytes retrieved, and fertilization and clinical PRs per embryo transfer were similar in HIV infected women and controls, live birth rates per embryo transfer were significantly lower for HIV infected women (6% versus 24%, *p* = 0.04) (Nurudeen et al. 2013 [23]). Unfortunately, cancelation rates were not compared between groups.

### 3.6. Tubal Disease

In four of the studies reviewed, the incidence of tubal disease could be compared between the study and control groups and in all of them a higher proportion of tubal disease in HIV infected women was documented, confirming previous reports on the literature [25] (Table 1). In two of them, a statistically significant difference was observed (Santulli et al. 2011 [22] and Nurudeen et al. 2013 [23]).

### 3.7. Number of Embryos Replaced per Transfer

In the studies analyzed, different criteria were adopted regarding embryo transfer (Table 2). In three of them, the authors reported they were more likely to replace a lower number of embryos in infected women (Terriou et al. 2005 [16]; Martinet et al. 2006 [18]; Santulli et al. 2011 [22]). However, documentation of a significant reduction in the mean number of embryos transferred was seen in only two of these (Martinet et al. 2006 [18]; Santulli et al. 2011 [22]).

## 4. Discussion

The variability of results observed in the studies analyzed can be mostly related to the small size of the samples studied, heterogeneity in study design, and methodology and incomplete control over potential confounding data. These limitations do not allow for the implementation of a statistical approach that might lead to solid conclusions, such as a meta-analysis.

Variables that are traditionally known to influence IVF/ICSI outcome and should therefore be controlled for are the following:

1. Female age.
2. Ovarian reserve.
3. Race/ethnicity.
4. Body Mass Index (BMI).
5. Tobacco consumption.
6. Tubal disease.
7. Number of embryos replaced.

A summary of the variables controlled per study is available in Table 3 and aspects to be considered regarding each of them are as follows.

### 4.1. Female Age

Population studies from areas where no consistent methods of birth control are applied show that natural fertility starts to decline after the age of 30, has its decline accelerated in the mid-30s, and ends at a mean age of 41 years [26]. The age-related effect on female fertility has also been shown in numerous reports on the results of IVF treatments due to a progressive decline on oocyte quality and quantity. The implantation rate per embryo clearly decreases after the age of 35 and the same has been shown for the probability of a live birth in IVF [27].

Due to this knowledge, control over female age is of paramount importance in any study on IVF treatment outcome. Such was not seen in three of the ten studies included in this review.

### 4.2. Ovarian Reserve

ART requires controlled ovarian stimulation for the achievement of improved efficacy. Currently, it is not consensual that HIV infection affects ovarian reserve. Seifer et al. evaluated the markers of ovarian follicular reserve and reproductive ageing in 187 HIV infected women not diagnosed as infertile [28]. Early follicular phase FSH, estradiol, inhibin B, and anti-Mullerian hormone (AMH) levels were measured. No evidence was found that HIV infection affects ovarian ageing. On the other hand, Ohl et al. measured serum FSH, inhibin B, AMH, and the antral follicle count (AFC) in 78 HIV infected women [29]. Mean FSH was 36% higher than that seen in the control group, whereas mean inhibin B and AMH were 57% and 23% lower, respectively. AFC was also significantly lower in the study group.

The possible influence of combined antiretroviral therapy on ovarian reserve and ovarian response to stimulation is not clear either. Side effects of the use of antiretroviral drugs such as mitochondrial dysfunction or modification in the lipid metabolism and insulin resistance could have consequences on folliculogenesis and ovulation regulatory processes [2, 30]. Oocytes from infertile HIV infected women on combined antiretroviral therapy were reported to have 32% depletion in mtDNA in comparison to infertile uninfected controls (*p* < 0.05) and depletion was even stronger in patients who failed to become pregnant in IVF treatments [31].

### 4.3. Race/Ethnicity

A growing number of studies have investigated the association between race/ethnicity and ART outcomes. Most of the studies have focused on comparisons between white Caucasian and black women and some have identified that the last group is more likely to have a diagnosis of tubal factor infertility, leiomyoma/uterine factor infertility, a longer duration of infertility before ART, and higher miscarriage/stillbirth rates [32–36]. Race/ethnicity as a risk factor for poor ART outcomes has been consistently acknowledged in recent large studies, even after adjusting for many confounding factors [37]. This may certainly be a source of bias for data collected in this review.

### 4.4. Body Mass Index (BMI)

Overweight and obesity are well-described risk factors for infertility, particularly as they relate to ovulation disorders. In spite of conflicting results of studies regarding the effect of high BMI on ART outcome, a systematic review and meta-analysis published in 2011 showed that overweight or obese women (BMI ≥ 25) had significantly lower clinical pregnancy (RR = 0.90, 95% CI 0.85–0.94) and live birth rates (RR = 0.84, 95% CI 0.77–0.92) and a significantly higher miscarriage rate (RR = 1.31, 95% CI 1.18–1.45) [38]. The analysis of overweight women alone (BMI ≥ 25–29.9) also showed lower clinical pregnancy (RR = 0.91, 95% CI 0.86–0.96) and live birth rates (RR = 0.91, 95% CI 0.85–0.98) and higher miscarriage rate (RR = 1.24, 95% CI 1.13–1.35), compared to women with BMI < 25. In conclusion, increased BMI is associated with adverse outcomes in women undergoing IVF/ICSI treatment, including lower live birth rates.

Increasing overweight and obesity in the HIV infected population were not seen until two decades ago. This relatively new reality has been attributed to several factors, such as better health due to combined antiretroviral therapy and improved life expectancy, which leads to obesity trends similar to those seen in the general population [39]. This parameter was not properly controlled for in the majority of studies analyzed here.

### 4.5. Tobacco

Over the last few decades, the prevalence of cigarette smoking among women of reproductive age has increased, reaching, in 2006, 33% in Europe and 28% in the USA [40], and is highly prevalent among persons infected with HIV [41]. Studies on IVF showed that cigarette smoking has deleterious effects on many aspects of treatment: ovarian responsiveness to gonadotropins, number of oocytes retrieved, fertilization, implantation, and early placentation [42].

### 4.6. Tubal Disease

In the studies reviewed, a higher proportion of tubal disease in HIV infected women was documented, confirming previous reports on the literature [25]. It is important to notice that many studies collected data at times during which it was still not a standard procedure to remove hydrosalpinx before IVF, which may have reduced live birth rates and constituted a source of bias [43].

Another factor associated with tubal disease that should be considered is the personal history of pelvic inflammatory disease. This condition has been shown to reduce ovarian response to stimulation due to direct damage to the ovaries and follicle loss or due to mechanical alterations of follicular development [18].

### 4.7. Number of Embryos Replaced per Transfer

The rate of preterm delivery and multiple gestations after ART is of particular concern for HIV infected women. Preterm labor or premature rupture of membranes may increase the risk of HIV vertical transmission [44]. Elective single embryo transfer for HIV infected women shall be considered in order to reduce the risk of multiple gestations [15]. A lower number of embryos replaced in HIV positive women may obviously contribute to lower pregnancy and live birth rates per transfer.

## 5. Conclusions

Data on PR in IVF/ICSI treatments performed in couples with HIV infected women are conflicting and it is still not clear if these patients display worse clinical outcomes per cycle initiated when compared to the general population. The same can be said specifically about ovarian response to stimulation, though a tendency for higher cancellation rates seems to be observed.

The small size of the samples studied and heterogeneity in study design and methodology make it difficult to draw clear conclusions about the impact of HIV infection in women on IVF outcome. Incomplete control over confounding variables (such as age, race/ethnicity, BMI, tobacco consumption, tubal disease, history of pelvic inflammatory disease, duration of infertility, and the number of embryos replaced) is of special concern. Altogether, such limitations hamper the performance of a proper meta-analysis.

In the future, prospective matched case-control studies are needed to understand the specific effects of HIV infection on ovarian response and ART outcome. Available data suggest some impact of HIV infection on ovarian function and IVF outcome, but noncontrolled sources of bias in published studies do not allow for definitive conclusions.

## Figures and Tables

**Table 1:** Summary of the published studies on fresh nondonor oocyte IVF/ICSI cycles in serodiscordant couples with an HIV infected female partner and baseline characteristics of study and control groups.

Study	Type of study	Treatment cycles/couples (*n*)	Control group	Mean female age	Body Mass Index		* *Tubal disease (%)		Markers of ovarian reserve	
Ohl et al. 2003 [14]	Clinical prospective	12/9	49 cycles HIV noninfected women with HIV infected male partners	35.9	Study NM	Control NM	Study NM	Control NM	Study Day 3 FSH = 9 IU/L	Control Day 3 FSH = 7 IU/L^*∗*^

Ohl et al. 2005 [15]	Clinical prospective	62/50	NA	35	NM	NA	32%	NA	Day 3 FSH = 7.1 IU/L	NA

Terriou et al. 2005 [16]	Age-matched cohort	66/29	66 age-matched cycles HIV noninfected women undergoing ICSI during the same period	35.8	NM	NM	30%	18%	Day 3 FSH = 7.4 IU/L	Day 3 FSH = 7.3 IU/L

Coll et al. 2006 [17]	Age-matched cohort	50/35	100 age-matched cycles HIV noninfected women with HIV noninfected male	NM	NM	NM	NM	NM	NM	NM

Martinet et al. 2006 [18]	Retrospective case-matched control	27/27	77 cycles HIV noninfected women matched for age, etiology and duration of infertility, history of pelvic surgery, and type of pituitary inhibition	35.5	NM	NM	70.8%	68.83%	NM	NM

Manigart et al. 2006 [19]	Prospective cohort	56/33	62 cycles Non-HIV infected women with HIV infected male partners	35.6	NM	NM	NM	NM	NM	NM

Douglas et al. 2009 [20]	Retrospective age-matched cohort	29/14	42 age-matched cycles HIV noninfected women undergoing ICSI for male factor infertility	36.5	NM	NM	35%	NM	Day 2 FSH = 7.7 IU/L	Day 2 FSH = 6.9 IU/L

Prisant et al. 2010 [21]	Retrospective case-matched control	94/52	94 cycles HIV noninfected women matched for age, etiology of infertility, rank of oocyte retrieval, and type of ART	34.7	NM	NM	NM	NM	NM	NM

Santulli et al. 2011 [22]	Prospective age-matched cohort	57/57	171 age-matched cycles HIV noninfected women with HIV noninfected male partners	34.2	24.2	22.9^*∗*^	Unilateral defect 8% Bilateral defect 54%	4,6% 25.2%^*∗*^	Day 3 FSH 6.5 IU/L AFC: 12.9	Day 3 FSH 7.1 IU/L AFC 13.9

Nurudeen et al. 2013 [23]	Retrospective age-matched cohort	<35 y: 8 ≥35 y: 52/36	8 age-matched cycles 52 age-matched cycles HIV noninfected controls undergoing treatment for male factor infertility	37.7	<35 y: 21 ≥35 y: 28	24.4 24.7	<35 y: 43% ≥35 y: 32%	20% 0%^*∗*^	<35 y: FSH = 6.8 IU/L AMH: NM ≥35 y: FSH = 8.5 IU/L AMH: 0.92 ng/mL	<35 y: FSH = 5.3 IU/L AMH: NM ≥35 y: FSH = 7.7 IU/L AMH: 0.6 ng/mL

^*∗*^ *p* value < 0.05. NA: not available. NM: not mentioned.

**Table 2:** Summary of ovarian stimulation and IVF outcomes of study and control groups.

Study	Cancellation rate (%)		Mean gonadotrophin dose/stimulation (IU)		Mean number of oocytes/retrieval		Mean number of embryos replaced		Implantation rate		PR/cycle (%)	PR/ET (%)	
	Study	Control	Study	Control	Study	Control	Study	Control	Study	Control	Study	Study	Control
Ohl et al. 2003 [14]	NM	NM	2893	1972	8.6	9	1.9 ± 0.6	1.7 ± 0.5	NM	NM	6.7	9.1	48.8^*∗*^
Ohl et al. 2005 [15]	9,7	NA	2793	NA	8.4	NA	1.8 ± 0.6	NA	11.8	NA	15.8	23.9	NM
Terriou et al. 2005 [16]	15,2	4.9^*∗*^	2898	2429^*∗*^	10.6	8.3	2	2.4	NM	NM	16.1	NM	NM
Coll et al. 2006 [17]	26	20	3721	3743	NM	NM	NM	NM	NM	NM	12	16.2	37.5^*∗*^
Martinet et al. 2006 [18]	18.5	14.29	4200	3348	6.55	8.27	1.3	1.94^*∗*^	NM	NM	11	14	24
Manigart et al. 2006 [19]	42.9	24.2	NM	NM	6	8	NM	NM	NM	NM	16.1	NM	NM
Douglas et al. 2009 [20]	17.2	NM	NM	NM	11.2	12.4	2.7 ± 0.3	3 ± 0.2	15	19	24.1	33	NM
Prisant et al. 2010 [21]	5.7	4.9	NM	NM	6.4	7.3	1.96 ± 0.5	1.94 ± 0.3	10.8	7.1	15.96^*∗∗*^	17.65	11,5
Santulli et al. 2011 [22]	16.2	9.5	2408	2283	8.3	9.6	1.5 ± 0.7	1.9 ± 0.7^*∗*^	21.9	27.4	NM	26.3^*∗∗*^	36.3
Nurudeen et al. 2013 [23]	20	NM	<35 y: 2105 ≥35 y: 3677	3103 4318	<35 y: 13.7 ≥35 y: 10.7	20.3 11.1	<35 y: 2.6 ± 0.8 ≥35 y: 2.7 ± 1.5	2.1 ± 0.6 2.9 ± 1.7	NM	NM	NM	<35 y: 63 ≥35 y: 17	57 25

^*∗*^ *p* value < 0.05. ^*∗∗*^PR per oocyte retrieval. NA: not available. NM: not mentioned.

**Table 3:** Summary of the variables that influence IVF/ICSI outcome which were controlled in each study.

Study	Female age	Ovarian reserve	Race/ethnicity	Body Mass Index	Tobacco consumption	Tubal disease	Number of embryos replaced per transfer
Ohl et al. 2003 [14]	—	Yes	—	—	—	—	Yes
Ohl et al. 2005 [15]	—	Yes	—	—	—	Yes	Yes
Terriou et al. 2005 [16]	Yes	Yes	—	—	—	Yes	Yes
Coll et al. 2006 [17]	Yes	—	Yes	—	—	—	—
Martinet et al. 2006 [18]	Yes	—	—	—	—	Yes	Yes
Manigart et al. 2006 [19]	—	—	—	—	—	—	—
Douglas et al. 2009 [20]	Yes	Yes	—	—	—	Yes	Yes
Prisant et al. 2010 [21]	Yes	—	—	—	—	—	Yes
Santulli et al. 2011 [22]	Yes	Yes	—	Yes	Yes	Yes	Yes
Nurudeen et al. 2013 [23]	Yes	Yes	Yes	Yes	Yes	Yes	Yes
